# Ireland's Early Provision for the Sick

**Published:** 1921-06-25

**Authors:** 


### Ireland's Early Provision for the Sick.

Ireland may be regarded as providing one of the earliest known hospitals. It is recorded in Sir Henry Burdett's "Hospitals and Asylums of the World" that, though the Hospital of St. John in Jerusalem may be looked upon as the earliest military hospital, there is evidence that long before the Cross became a sacred symbol such an institution was in existence. In 300 B.C. the Palace of Emania was founded in Ireland by the Princess Macha "of the golden hair," and continued to be the chief royal residence in Ulster till it was destroyed in 332 A.D. To this were attached two houses, in one of which the Red Branch Knights hung up their arms and trophies, while in the other the sick were cared for and the wounded healed. The latter bore the name of "Broin Bearg," the House of Sorrow. This example of the princess was widely followed throughout Ireland, and the ancient laws of that country sanctioned the providing for the sick "a physician, food, proper bed, furniture, and a proper house."

